# Mutations in *ALDH6A1* encoding methylmalonate semialdehyde dehydrogenase are associated with dysmyelination and transient methylmalonic aciduria

**DOI:** 10.1186/1750-1172-8-98

**Published:** 2013-07-09

**Authors:** Julien L Marcadier, Amanda M Smith, Daniela Pohl, Jeremy Schwartzentruber, Osama Y Al-Dirbashi, Jacek Majewski, Sacha Ferdinandusse, Ronald JA Wanders, Dennis E Bulman, Kym M Boycott, Pranesh Chakraborty, Michael T Geraghty

**Affiliations:** 1 Department of Genetics, Children’s Hospital of Eastern Ontario, Ottawa ON, Canada; 2 Ottawa Hospital Research Institute, Ottawa, ON, Canada; 3 Department of Pediatrics, Division of Neurology, Children’s Hospital of Eastern Ontario, University of Ottawa, Ontario, Canada; 4 McGill University and Genome Quebec Innovation Centre, Montreal, QC, Canada; 5 Newborn Screening Ontario, Children’s Hospital of Eastern Ontario, Ottawa, ON, Canada; 6 Children’s Hospital of Eastern Ontario Research Institute, University of Ottawa, Ottawa, ON, Canada; 7 Laboratory Genetic Metabolic Diseases, Academic Medical Center, University of Amsterdam, Amsterdam, The Netherlands; 8 Department of Pediatrics, Division of Metabolics, Children’s Hospital of Eastern Ontario, University of Ottawa, Ottawa, ON K1H8L1, Canada

**Keywords:** Methylmalonate semialdehyde dehydrogenase, ALDH6A1, Methylmalonic acid, Delayed myelination, Whole exome sequencing

## Abstract

**Background:**

Methylmalonate semialdehyde dehydrogenase (MMSDH) deficiency is a rare autosomal recessive disorder with varied metabolite abnormalities, including accumulation of 3-hydroxyisobutyric, 3-hydroxypropionic, 3-aminoisobutyric and methylmalonic acids, as well as β-alanine. Existing reports describe a highly variable clinical and biochemical phenotype, which can make diagnosis a challenge. To date, only three reported cases have been confirmed at the molecular level, through identification of homozygous mutations in *ALDH6A1*, the gene encoding MMSDH. Confirmation by enzyme assay has until now not been possible, due to the extreme instability of the enzyme substrate.

**Methods and results:**

We report a child with severe developmental delays, abnormal myelination on brain MRI, and transient/variable elevations in lactate, methylmalonic acid, 3-hydroxyisobutyric and 3-aminoisobutyric acids. Compound heterozygous mutations were identified by exome sequencing and confirmed by Sanger sequencing within exon 6 (c.514 T > C; p. Tyr172His) and exon 12 (c.1603C > T; p. Arg535Cys) of *ALDH6A1*. The resulting amino acid changes, both occurring in residues conserved among mammals, are predicted to be damaging at the protein level. Subsequent MMSDH enzyme assay demonstrated reduced activity in patient fibroblasts, measuring 2.5 standard deviations below the mean.

**Conclusions:**

We present the fourth reported case of MMSDH deficiency with confirmation at the molecular level, and expand on what is already an extremely variable clinical and biochemical phenotype. Furthermore, this is the first report to demonstrate a corresponding reduction in MMSDH enzyme activity. This report illustrates the emerging utilization of whole exome sequencing and variant data filtering using clinical data as an early tool in the diagnosis of rare and variable conditions.

## Background

**Figure 1:**
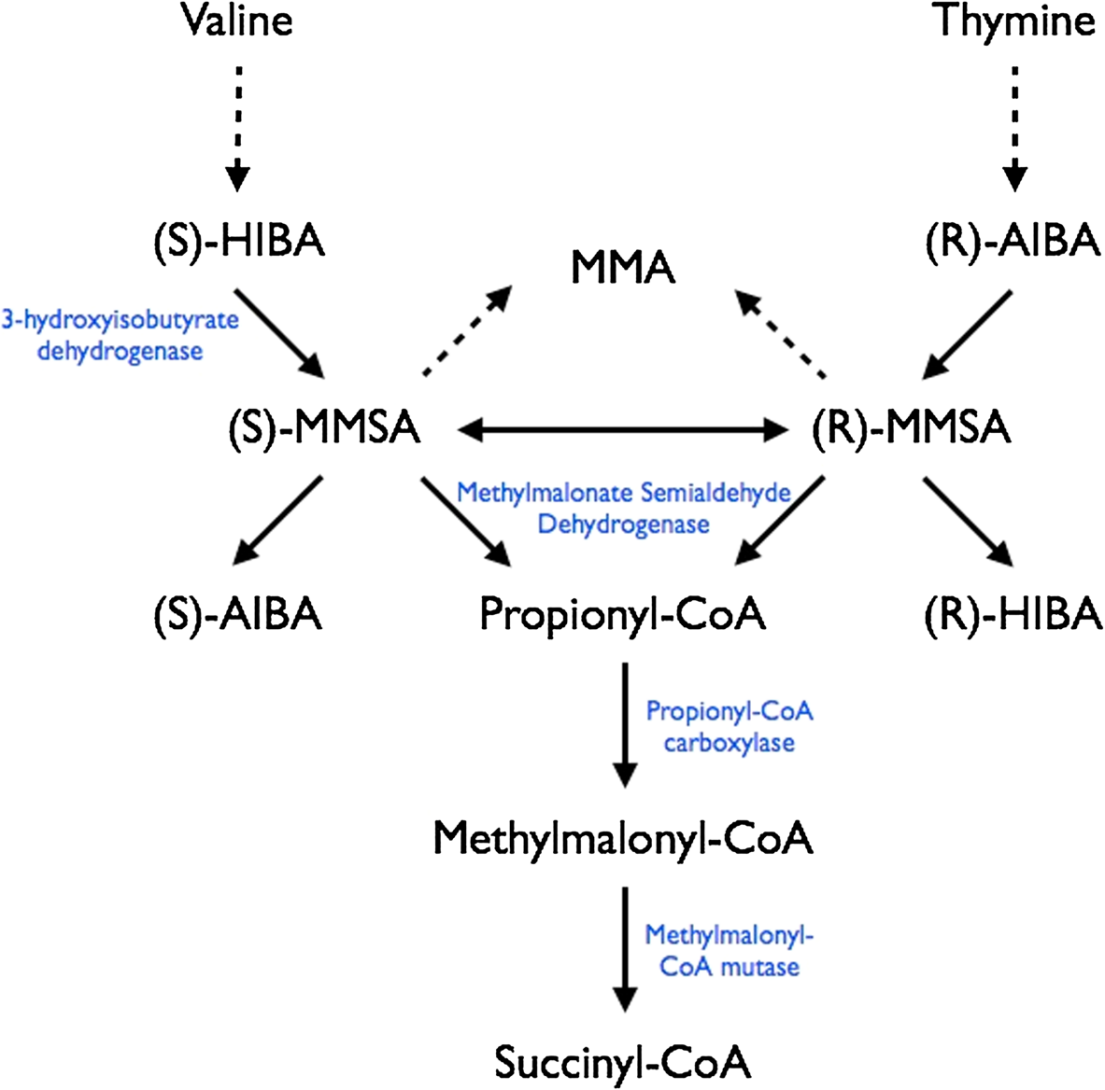
**Valine and thymine catabolism, resulting in the oxidative decarboxylation of methylmalonic semialdehyde (MMSA) into propionyl-CoA by methylmalonate semialdehyde dehydrogenase.** HIBA: 3-hydroxyisobutyric acid; AIBA: 3-aminoisobutyric acid.

Methylmalonate semialdehyde dehydrogenase (MMSDH) is involved in the catabolic breakdown of both valine and thymine (MIM#603178). Metabolism of valine produces the intermediate (S)-3-hydroxyisobutyric acid (HIBA), which is oxidized to (S)-methylmalonic semialdehyde (MMSA) by 3-hydroxyisobutyrate dehydrogenase. Thymine metabolism generates (R)-aminoisobutyric acid (AIBA), which is then deaminated to (R)-methylmalonic semialdehyde. These two enantiomers of MMSA are substrates for MMSDH, which catalyzes their oxidative decarboxylation to propionyl-CoA (Figure 1). Very few case reports have described individuals with MMSDH deficiency, and all have been in the context of elevated urine levels of 3-hydroxyisobutyric acid, among other metabolic abnormalities. The initial report by Pollitt et al. [1] describes an asymptomatic child ascertained due to high methionine levels on newborn screening, who was found to have 3-hydroxyisobutyric aciduria, and ultimately shown to carry a homozygous missense mutation (c.1336G > A) in *ALDH6A1*, the gene encoding MMSDH [2]. A recent report described two unrelated children with 3-hydroxyisobutyric aciduria and different, novel homozygous missense mutations in *ALDH6A1*[3]. Neither report could demonstrate a reduction in MMSDH enzyme activity, given the unstable behaviour of methylmalonate semialdehyde in enzyme assays. As such, the discovery of deleterious mutations in *ALDH6A1* has until recently served as the only means of diagnosing MMSDH dysfunction.

In this report, we describe a young female child with severe developmental delays, abnormal brain MRI and transient elevations in methylmalonic acid (MMA), in addition to raised levels of 3-hydroxyisobutyrate and plasma lactate. Exome sequencing revealed novel compound heterozygous missense mutations in *ALDH6A1,* with subsequent studies in fibroblasts demonstrating reduced MMSDH enzyme activity. This is the fourth case of a child with deficiency of MMSDH due to mutations in *ALDH6A1*, and the first such case to concurrently demonstrate a deficiency at the enzyme level. This report expands our understanding of the biochemical and clinical aspects of this very rare condition, and highlights that it may in fact be significantly under-diagnosed, given that many of the biochemical abnormalities initially observed in our patient had normalized by the age of 18 months.

## Patient and methods

Institutional research ethics board approval (Children’s Hospital of Eastern Ontario) was obtained prior to this study. Parents of the patient provided informed consent for exome sequencing as well as permission to publish clinical information and images contained within this report.

### Clinical description

The patient (AB), now 36 months of age, was initially referred at 7 months for developmental delays, dysmorphic features, and slightly raised urine and plasma methylmalonic acid. She was born at 40 + 5 weeks gestation to non-consanguineous parents following an uneventful pregnancy and delivery. Birth weight was 3657 g (75th percentile). She was found to be hypotonic and have visual fixation and tracking difficulties at 3 months of age, thereby initiating clinical investigation. By 19 months of age she had made slow progress, but continued to show global developmental delay without regression. She could sit with assistance, but was unable to crawl or pull to stand. Vocalizations consisted of monosyllabic noises. She has never had a seizure-like episode. Family history is significant only for a paternal cousin with congenital deafness.

Initial physical examination revealed normal growth parameters (head circumference 50th percentile, length 75th percentile, weight 90-95th percentile). However, interval growth in head circumference has not adequately progressed, and at the time of this report was at the 10th percentile. Her face had a square shape, with slight frontal bossing, a tall forehead, and sparse hair temporally. Ocular findings included bilateral epicanthic folds, mild hypertelorism, and bilateral mottled retinal pigmented epithelium. The nose was short and anteverted, with a broad, flat base, and a short philtrum. Her mouth was tented and she had a high-arched palate. Halluces were broad bilaterally, and a single palmar crease was noted on the right palm. Axial tone was markedly decreased, with fluctuating peripheral tone. Deep tendon reflexes were brisk, but there was no ankle clonus. Muscle bulk and strength were normal, and sensation was grossly intact. Coordination was inappropriate for her age, and her movements were dystonic.

**Figure 2:**
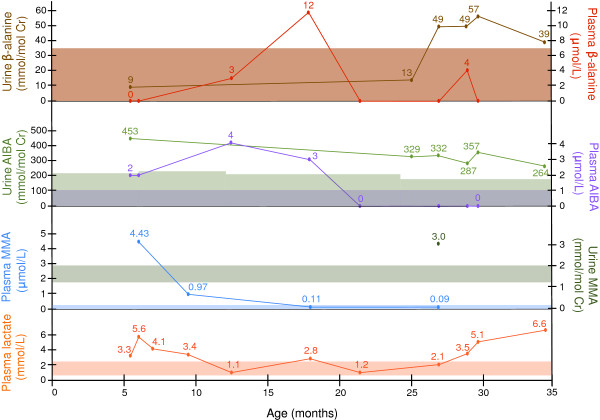
**Chronological outline of select investigations, with fluctuating levels over time.** The variability is independent of dietary modifications or pharmacological treatment. Normal ranges are represented by shaded areas. AIBA: 3-aminoisobutyric acid.

Investigations at 6 months showed increased urinary excretion of MMA, peak plasma MMA levels of 4428 nmol/L (normal: 0–270 nmol/L), and plasma lactate levels of 5.6 mmol/L (normal: 0.5 - 2.2 mmol/L). These and other metabolite levels have fluctuated over time, without intervention and independent of clinical status. More recent measurements, taken while the child has been well, have shown elevated plasma lactate concentrations, with a concomitant rise in urinary MMA and β-alanine, as well as persistently elevated urinary aminoisobutyric acid (Figure 2). Although not initially recognized, urine organic acid GCMS profiles showed elevated 3-hydroxybutyric acid. Subsequent ion extraction for m/z 191 and m/z 177 separated the peak into 3-hydroxybutyrate and 3-hydroxyisobutyrate, isomers that typically co-elute as a single peak, respectively. The latter was present in concentrations approximately 10-fold larger than the former. Additionally, methylcitrate and 3-hydroxypropionate were absent. Urine amino acids demonstrated increased levels of aminoisobutyrate (maximum 453 nmol/mol creatinine at 6 months; normal: 33–170 nmol/mol creatinine) and β-alanine (maximum 57 mmol/mol creatinine at 29 months; normal: 0–7 mmol/mol creatinine) (Figure 2). Plasma alanine levels have remained within normal limits (ranging from 207 to 375 μmol/L), with a mild elevation to 646 μmol/L that corresponded with the lactate level of 5.1 at 29 months. Plasma ammonium, creatine kinase, liver function tests and thyroid studies were all normal. Early acylcarnitine profiles twice showed small elevations in C3 (maximum 0.76 μmol/L; normal: < 0.65 μmol/L) and C10 (maximum 0.36 μmol/L; normal: < 0.26 μmol/L) acylcarnitines, which have since normalized. Quantitative plasma carnitine (free and total), very long chain fatty acids, transferrin isoelectric focusing, biotinidase, and 7-dehydrocholesterol were all normal. Cerebrospinal fluid lactate, amino acids and neurotransmitters were normal, as were CSF organic acids when compared to published control values [4]. Serum vitamin B12 and plasma homocysteine levels were normal, and cultured fibroblasts showed normal propionate, methyltetrahydrofolate and cyanocobalamin incorporation. While a mitochondrial respiratory chain defect was suspected, muscle histology, immunohistochemistry and electron microscopy were normal. Additionally, respiratory chain studies in frozen muscle and skin fibroblasts were all normal.

**Figure 3:**
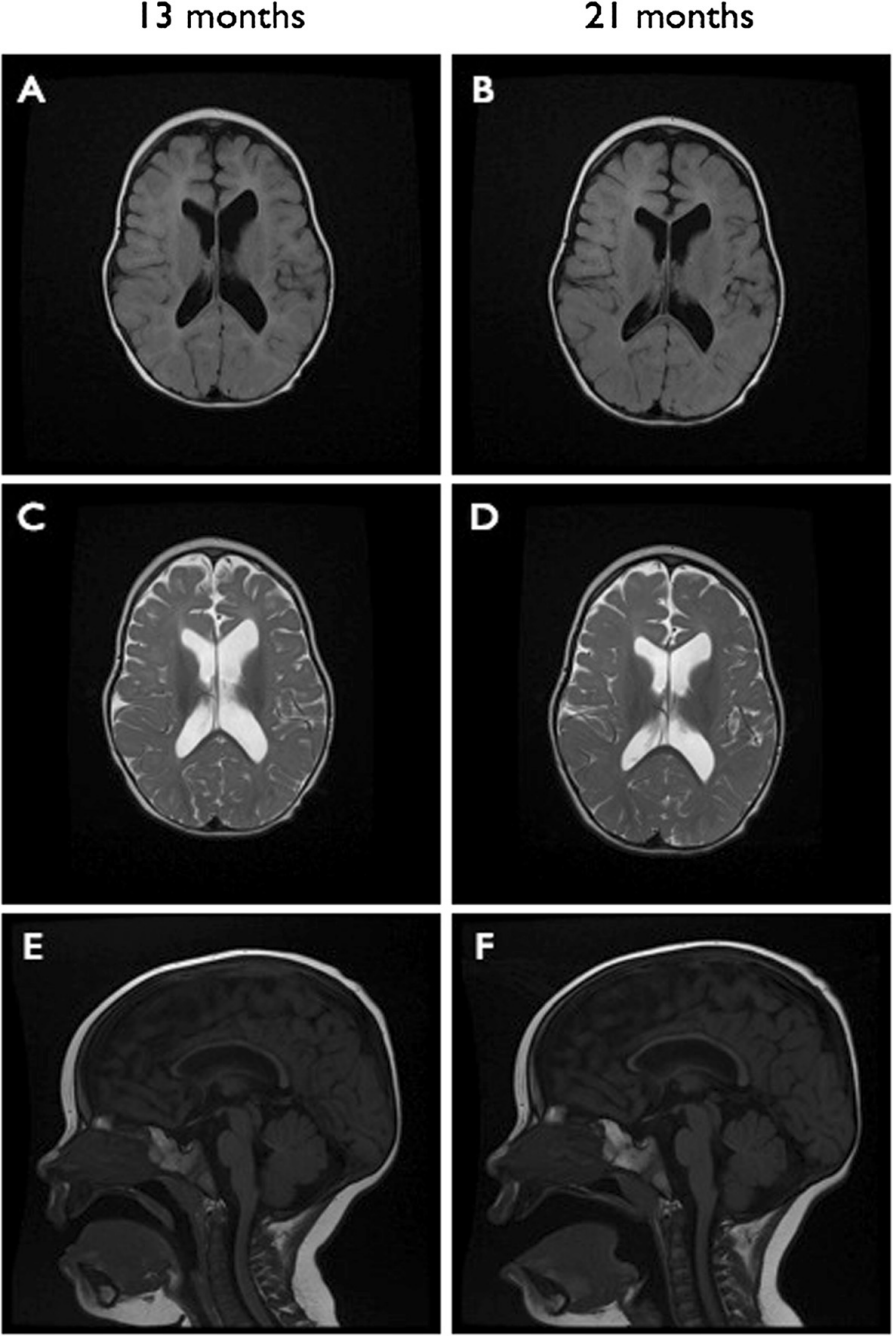
**MRI images taken at 13 and 21 months of age. (A, B)** Axial FLAIR images show only minimal interval progression in myelination over a 9 month span, with diffuse thinning of periventricular white matter evident at 13 months. **(C, D)** Axial T2-weighted images show markedly delayed myelination, with mild ventricular dilatation and slight enlargement of the frontal extra-axial spaces. **(E, F)** Sagittal T2-weighted images show a diffusely thin corpus callosum. MR spectroscopy demonstrated normal peaks of NAA, creatine and choline, and absence of a lactate peak.

Brain MRI at 13 and 21 months of age demonstrated a thin corpus callosum, diffuse delay in myelination, and very slow progression in myelination during the 8 month interval between images (Figure 3). Note was also made of asymmetrically enlarged lateral ventricles, with tiny subependymal gray matter heterotopia at the wall of the left lateral ventricle, and prominent extra-axial spaces bifrontally. MR spectroscopy demonstrated normal NAA, creatine, choline, and lactate peaks. Additional neurological studies have included visual evoked potentials (mildly increased latencies, but otherwise normal), as well as normal brainstem auditory evoked responses and nerve conduction studies.

Molecular genetic testing included normal CGH and SNP microarrays. Furthermore, DNA sequence analysis found no mutations in *SUCLA2* or *SUCLG1*, genes that encode the succinyl-CoA ligase complex and are associated with lactic acidosis, methylmalonic aciduria and mitochondrial depletion syndromes. Sequencing of *SUCLG2* was not performed.

A diagnosis was not made following several months of extensive investigations, though the collection of abnormal results were highly suggestive of either a defect in the metabolism of MMA or of mitochondrial dysfunction. It was at this point that whole exome sequencing was pursued, looking specifically for variants that could fall within these categories.

### Exome sequencing

We followed standard manufacturer protocols to perform target capture with the Agilent SureSelect All Exon 50 MB (V3) exome enrichment kit and sequencing of 100 bp paired end reads on Illumina HiSeq 2000, which generated 11.7 Gb of sequence for the sample. We removed adaptor sequences and quality trimmed reads using the Fastx toolkit [5] and then used a custom script to ensure that only read pairs with both mates present were subsequently used. Reads were aligned to hg19 with BWA 0.5.9 [6], and indel realignment was done using the GATK [7]. Duplicate reads were then marked using Picard [8] and excluded from downstream analyses. We assessed coverage of consensus coding sequence (CCDS) bases using the GATK, which showed that the sample had 92.3% of CCDS bases covered by at least 10 reads, and 87.8% of CCDS bases covered by at least 20 reads. Single nucleotide variants (SNVs) and short insertions and deletions (indels) were called using samtools mpileup [9] with the extended base alignment quality (BAQ) adjustment (−E), and were then quality filtered to require at least 20% of reads supporting the variant call. Variants were annotated using both Annovar [10] and custom scripts to identify whether they affected protein coding sequence, and whether they had previously been seen in dbSNP132 [11], the 1000 genomes dataset (Nov. 2011) [12], the NHLBI GO exomes [13], or in approximately 540 exomes previously sequenced at our center.

### Variant validation

Sanger sequencing was used to validate mutations identified by next-generation sequencing and to evaluate segregation of variants in the family. Blood samples were obtained and DNA was extracted from the unaffected parents and sibling. PCR was performed with primers 5′-GAAGGGCAAGTCAGTGTACC-3′ and 5′-CAACAAACATGGAGGTTAAAATG-3′ to test for the c.514 T > C variant and 5′-GATGGCTAAGGTTTGATTGTTTAC-3′ and 5′-GAAGAGCAAGTGAGAAATCTGG-3′ to test the c.1603C > T variant.

### Enzyme assay

Enzyme activity measurement was performed using methylmalonate semialdehyde as substrate in the presence of NAD^+^ and CoenzymeA. Because methylmalonate semialdehyde is an unstable compound, a diethyl acetal of methylmalonate semialdehyde was synthesized as described [14] and hydrolyzed as described [15] prior to performing the enzymatic assay. The product of the enzyme reaction, propionyl-CoA was converted to propionyl-carnitine by carnitine acetyltransferase (CRAT), which was detected by tandem-mass spectrometry using deuterium labelled propionyl-carnitine as internal standard (unpublished data, manuscript describing enzyme assay in preparation [16]). Reference values were determined by measuring MMSDH activity in 13 different cultured skin fibroblast cell lines from control subject.

## Results

Whole exome sequencing identified two novel variants in the gene *ALDH6A1*, supporting a diagnosis of MMSDH deficiency. A total of 32 variants that affect protein sequence or canonical splice sites while fitting a recessive disease model were identified. The only variants seen in the MitoCarta database (an inventory of 1013 nuclear and mtDNA genes encoding proteins with strong support of mitochondrial localization) [17], both occurred in *ALDH6A1*. A missense mutation in exon 12 (NM_005589, c.1603C > T), causing an arginine-to-cysteine change at the last amino acid before the stop codon (p. Arg535Cys), has only once been reported in the heterozygote state out of 6503 NHLBI exomes [13], and has not been seen in the 1000 Genomes dataset [12] or the 540 exomes from other rare disease projects studied by our sequencing facility. A second missense mutation in exon 6 (NM_005589, c.514 T > C), which produces a tyrosine-to-histidine change (p. Tyr172His), has not been reported in any of these datasets. Mutations were confirmed by Sanger sequencing. Additionally, each parent was confirmed to be a carrier of one of the mutations. The healthy sibling of the patient did not carry either mutation. Both affected amino acid residues are highly conserved among mammals. The resulting amino acid changes are predicted to be damaging by SIFT [18] (scores 0 and 0.01, respectively) and Polyphen2 [19] (scores 0.986 and 0.838, respectively). Further examination of the exome sequencing results identified no rare variants in genes known to be associated with hypomyelination, including *PLP1, GJC2, AIMP1, HSP60, FAM126A, SLC16A2, LMNB1, SLC17A5 and L1CAM*. Despite extensive investigations, no other cause was identified for this child’s phenotype. In contrast, exome sequencing does not make prior assumptions of causation, and thus finding two predicted pathogenic mutations in *ALDH6A1*, a gene that can be reasonably related to the biochemical findings, is strongly suggestive of disease causation.

Fibroblast studies at the Laboratory of Genetic Metabolic Diseases (Academic Medical Center, Amsterdam, The Netherlands) revealed reduced activity of MMSDH. Measured activity was 36 pmol/(min.mg protein) (normal range 51–184; mean 117; standard deviation ±33).

## Discussion

MMSDH deficiency is an extremely rare, autosomal recessive disorder of valine and thymine metabolism. To date, mutations in *ALDH6A1* have only been identified in three individuals (summarized in Table 1). In the first such case, the patient presented incidentally with hypermethioninemia on his newborn screen [1,20]. The raised methionine could not be related to the subsequent discovery of persistent urine and plasma abnormalities in the form of elevated β-alanine, 3-hydroxypropionic, 3-aminoisobutyric, 3-hydroxyisobutyric and (S)-2-(hydroxymethyl)butyric acids. The child has remained medically and developmentally well as of 4 years of age. Molecular analysis by Chambliss et al. [2] identified a homozygous missense mutation (c.1336G > A; p. Gly446Arg) in the *ALDH6A1* gene that has 100% conservation of the glycine residue in vertebrates, and has not been seen in the 6503 NHLBI exomes. The remaining two molecularly confirmed cases were reported by Sass et al. [3]. One child was a European boy born to consanguineous parents, who presented with elevated 3-hydroxyisobutyric acid and mild elevations in 3-hydroxypropionic and 2-ethyl-3-hydroxypropionic acids. He had mild developmental delay and dysmorphisms, and passed away at 26 months of age following acute hepatoencephalopathy secondary to liver failure. Postmortem analysis showed cerebral edema with white matter vacuolization and microcalcifications in the frontal cortex, not previously seen on imaging while alive. He was found to be homozygous for a c.785C > A (p. Ser262Tyr) change in *ALDH6A1*. The second case was a young girl of Pakistani origin, previously described by Shield et al. [21]. She was the product of a consanguineous union, with severe developmental delays, dysmorphic features, microphthalmia, cataracts and postnatal microcephaly. Brain MRI at 1 year of age showed significantly delayed myelination, thinning of the corpus callosum and microcephaly. Urine organic acids showed elevated 3-hydroxyisobutyric and 3-hydroxypropionic acid levels. Lactate levels were only mildly elevated. She was homozygous for a c.184C > T (p. Pro62Ser) mutation in *ALDH6A1*. Of note, the child’s older brother was also found to have increased levels of 3-hydroxyisobutyric acid without lactic acidosis, though molecular analysis was not reported. His phenotype was on the milder end of the spectrum, with microcephaly and moderate learning difficulties. MRI of his brain showed only isolated microcephaly. The mutations reported in both the European and Pakistani cases occurred in highly conserved amino acids, and were predicted to be disease causing. The variable clinical and biochemical spectrum demonstrated by these three cases, in addition to the one reported here (Table 1), illustrates the need for detailed genotype-phenotype correlation studies for patients carrying *ALDH6A1* mutations.

**Table 1:** Clinical and biochemical summary of molecularly confirmed patients in the literature

	**Our patientt**	**Pollitt et al.**[1]	**Shield et al.**[21]	**Sass et al.**[3]
		**Gray et al.**[20]	**Sass et al.**[3]	
		**Chambliss et al.**[2]		
**Patient information**	Female	Male	Female	Male
	Mixed European ancestry	Pakistani ancestry	Pakistani ancestry	Mixed European ancestry
	Parents non-consanguineous	Consanguinity not specified	Parents consanguineous	Parents consanguineous
***ALDH6A1 *mutations**	c.514T > C	c.1336G > A	c.184C > T	c.785C > A
	c.1603C > T	c.1336G > A	c.184C > T	c.785C > A
**Biochemical abnormalities**	↑ plasma/urine MMA	↑ methionine	↑ 3-hydroxypropionate	
	↑ lactate	↑ 3-hydroxyproprionate	↑ methylcitrate	Mild ↑ 3-hydroxypropionate
	↑ HIBA	↑ HIBA	Mild ↑ lactate	↑ HIBA
	↑ AIBA	↑ AIBA	↑ HIBA	↑ β-alanine
	↑ β-alanine	↑ β-alanine	↑ AIBA	
**CNS findings**	Severe developmental delays, dystonia and microcephaly.	Normal development	Severe developmental delays, hypotonia and microcephaly.	Early delays, corrected by 25 months. Relative microcephaly.
	Delayed myelination and thin corpus callosum on MRI	No imaging reported	Delayed myelination and thin corpus callosum on MRI	No imaging reported. Frontal cortex microcalcifications on autopsy.
**Clinical findings**	Tall forehead, epicanthal folds, mild hypertelorism, short philtrum, broad halluces, right single palmar crease.	Healthy	Microphthalmia and cataracts, diagnosed as Warburg Micro Syndrome. Narrow, downslanting palpebral fissures, short nose, depressed nasalbridge.	Bulbous nose, long philtrum.
		No reported dysmorphisms		Died at 26 months from hepatoencephalopathy and liver failure following a febrile illness.

*HIBA*: 3-hydroxyisobutyric acid; *AIBA*: 3-aminoisobutyric acid.

Other cases of 3-hydroxyisobutyric aciduria with metabolic profiles suggestive of MMSDH deficiency have been reported with variable clinical and biochemical pictures [22-28]. Two of these cases had loading studies in cultured fibroblasts that were suggestive of deficient MMSDH enzymatic activity. The first was a male who presented at 6 years of age with failure to thrive, vomiting, lethargy and a metabolic acidosis [22,25]. Urine screen showed elevated levels of 3-hydroxyisobutyric acid and lactate. Physical examination found subtle dysmorphisms in the form of a small, triangular face with low-set ears, fifth finger clinodactyly, and 2/3 toe syndactyly. The second child was a male who at 22 months of age was found to have elevated methylmalonate and normal propionylcarnitine levels prior to surgery for gut malrotation and Meckel’s diverticulum repair [26]. He is otherwise globally delayed, and has never had an episode of metabolic acidosis. Notably, mutations in *ALDH6A1* could not be identified in either individual [2]. Demonstration of reduced MMSDH activity in fibroblasts was not available at the time of study.

As we have highlighted, the diagnosis of MMSDH deficiency is problematic. Case reports are exceedingly rare and the phenotype appears to be highly variable. This raises the issue of bias of ascertainment - a common complication of studying rare inborn errors of metabolism. Adding further to the difficulty of diagnosis is that the biochemical changes seen in our patient have been transient and varied. Of particular note, peak plasma MMA levels were reached when the child was approximately 6 months old, after which they progressively declined to the normal range (Figure 2). This was achieved without any intervention – dietary, pharmacologic or otherwise. Serum MMA levels decreased from 4428 nmol/L at 6 months to 90 nmol/L at 27.5 months. Plasma lactate levels had a prolonged period of normalcy after 18 months of age, with a recent increase to 6.6 mmol/L on our most recent investigations at 34.5 months. Our investigations failed to identify abnormalities in urine organic acids, and showed small elevations in aminoisobutyric acid and β-alanine in urine amino acids. One can speculate that the abnormal findings correspond to a peak time of growth with associated metabolite turnover. As in our case, mild elevations in 3-hydroxyisobutyric acid, aminoisobutyric acid and β-alanine may go unreported, or missed, due in part to the fact that 3-hydroxyisobutyric acid is isobaric to 3-hydroxybutyric acid. Elevations in 3-hydroxybutyrate are non-specific, and can be attributed to any process that might induce ketogenesis. As such, 3-hydroxyisobutyrate can be easily missed on mass spectrometry if one is not specifically looking for that compound. We therefore suggest that the finding of mild methylmalonic aciduria should prompt a careful search for metabolites related to MMSDH deficiency.

Previous reports of molecularly confirmed MMSDH deficiency have described various brain abnormalities, including postnatal microcephaly, cerebral microcalcifications and, in one case, acute hepatoencephalopathy associated with cerebral edema and death. We report hypoplasia of the corpus callosum and profoundly delayed myelination between 13 and 21 months of age (Figure 3). The patient described by Shield et al. [21] similarly showed delayed myelination at 1 year of age. It is ultimately not clear how deficiency of MMSDH would cause brain dysmyelination and developmental delays. One can speculate on the toxicity of the semialdehyde itself, as well as its related metabolites. It is interesting to note that MMSDH also plays a role in thymine catabolism, and that disorders of mitochondrial nucleotide pools are associated with mitochondrial depletion and encephalopathy. For example, as is seen in our case, MMA and lactic acidosis are also features of succinyl-CoA ligase deficiency. Further information may be gained in the future with the design of MMSDH knockouts in model systems, though as of this writing there are none available.

The place of exome sequencing in the clinic remains unclear. In our case, it was done after an extensive diagnostic workup. Certainly in this patient it could have prevented the need for a muscle biopsy. As well, it was the only diagnostic test available for this disease, as neither enzymology nor single gene testing were available. Furthermore, where clinical and laboratory information is available that points to specific pathways or organelles (such as MMA metabolism or mitochondrial disease), this information can be used to filter the sequencing data and increases the likelihood of finding a definitive answer, while simultaneously reducing the number of variants of unknown significance.

## Conclusions

We present the fourth molecularly confirmed case of MMSDH deficiency, caused by compound heterozygous mutations in the *ALDH6A1* gene, and the first such case to demonstrate a concomitant decrease in enzyme activity. This report serves to illustrate both the clinical and biochemical variability present in this rare condition, and highlights the importance of searching for specific metabolites in the context of elevated MMA levels. Also illustrated is the emerging utilization of whole exome sequencing coupled with predetermined variant filtering as an early tool in the diagnosis of rare and variable conditions. Finally, given the difficulties with diagnosis, we suspect this is an under-recognized disorder, and that our understanding of this rare condition will improve as more patients are identified.

## Abbreviations

MMSDH: Methylmalonate semialdehyde dehydrogenase; ALDH6A1: Aldehyde dehydrogenase 6 family member A1; MMA: Methylmalonic acid; SIFT: Sorting intolerant from tolerant; PolyPhen-2: Polymorphism phenotyping.

## Competing interests

The authors declare that they have no competing interests.

## Authors’ contributions

JLM and MTG wrote the manuscript. KMB and DB designed and coordinated the study. MTG, DP, OYA, PC and JLM provided subspecialist consultation services, serial clinical examinations and diagnostic testing. JS and JM carried out the analysis of the next-generation sequencing data. AMS and KMB arranged for Sanger sequencing of the *ALDH6A1* gene. RJAW and SF carried out biochemical testing on fibroblasts. All authors read and approved the final manuscript.
